# Meibomian Gland-Mediated Drug Delivery via Eyelid Application of Troxipide Nanoparticles Improves an N-Acetylcysteine-Induced Dry Eye

**DOI:** 10.3390/pharmaceutics18080973

**Published:** 2026-08-08

**Authors:** Hiroko Otake, Rie Tanaka, Fumihiko Ogata, Manju Misra, Kazutaka Kanai, Masanobu Tsubaki, Naoki Yamamoto, Naohito Kawasaki, Noriaki Nagai

**Affiliations:** 1 Faculty of Pharmacy, Kindai University, 3-4-1 Kowakae, Higashi-Osaka 577-8502, Osaka, Japan; hotake@phar.kindai.ac.jp (H.O.); 2633420006v@kindai.ac.jp (R.T.); ogata@phar.kindai.ac.jp (F.O.); kawasaki@phar.kindai.ac.jp (N.K.); 2 Department of Pharmaceutics and Pham. Technology, L. M. College of Pharmacy, Navrangpura, Ahmedabad, GJ 380009, India; manju.misra@lmcp.ac.in; 3 Department of Small Animal Internal Medicine II, School of Veterinary Medicine, Kitasato University, 35-1 Higashi 23 ban-cho, Towada 034-8628, Aomori, Japan; kanai@vmas.kitasato-u.ac.jp; 4 Laboratory of Pharmacotherapy, Kagawa School of Pharmaceutical Sciences, Tokushima Bunri University, 1314-1 Shido, Sanuki 769-2193, Kagawa, Japan; tsubaki@kph.bunri-u.ac.jp; 5 University Startup Promotion Center, Research Promotion Headquarters, Fujita Health University, 1-98 Dengakugakubo, Kutsukake-cho, Toyoake 470-1192, Aichi, Japan; naokiy@fujita-hu.ac.jp; 6 Department of Pharmaceutical Engineering, Faculty of Engineering, Sanyo-Onoda City University, 1-1-1 Daigaku-dori, Sanyo-Onoda 756-0884, Yamaguchi, Japan

**Keywords:** troxipide, nanoparticle, dry eye, eyelid, ocular drug delivery

## Abstract

**Background/Objectives:** Dry eye disease (DED) is a multifactorial disorder characterized by tear film instability, inflammation, and ocular surface damage, which significantly impairs visual function and quality of life. Conventional ophthalmic formulations, such as eye drops, have low bioavailability owing to rapid elimination, necessitating frequent administration. In this study, we developed an eyelid-applied drug delivery system (DDS) based on troxipide (TRO) nanoparticle formulation (TRO-NP@EG) to achieve sustained ocular surface delivery. **Methods:** TRO nanosuspensions were prepared by wet bead milling and incorporated into a Carbopol-based gel. Particle size, dispersion stability, and uniformity were evaluated, and in vitro drug release studies was compared with that of TRO-MP@EG. In vivo drug transfer into tear fluid was assessed in rabbits following eyelid application, and therapeutic efficacy was evaluated in an N-acetylcysteine-induced dry eye model. **Results:** TRO nanosuspensions had a mean particle size of approximately 118 nm. TRO-NP@EG exhibited superior dispersion stability and uniformity and achieved 2.5-fold higher drug release than TRO-MP@EG, while the nanoparticles remained in solid form. In vivo studies in rabbits, TRO-NP@EG significantly enhanced drug transfer into tear fluid, primarily via the meibum pathway. Furthermore, TRO-NP@EG significantly improved mucin levels, tear secretion, and tear film stability compared with TRO-MP@EG in an N-acetylcysteine-induced dry eye model. **Conclusions:** These findings suggest that eyelid application of nanoparticle-based formulations enables efficient and sustained drug delivery to the ocular surface via the meibomian glands. Therefore, TRO-NP@EG represents a promising therapeutic strategy for DED, providing enhanced efficacy and a novel route of administration for ophthalmic DDSs.

## 1. Introduction

Tears play multiple essential roles in maintaining normal ocular physiology, including protecting the corneal and conjunctival surfaces from the external environment and preventing desiccation of the ocular surface [1]. In addition, tears supply nutrients to the cornea and conjunctiva, remove foreign particles, exert antimicrobial activity against bacteria and viruses, and provide lubrication during blinking. Structurally, the tear film consists of three layers: an outer lipid layer, a middle aqueous layer, and an inner mucin layer [2]. These functions are maintained only when all three layers are present in an appropriate balance and composition; disruption of either the quantity or quality of any layer leads to tear film instability. Clinically, tear film breakup (TFB) time is assessed using fluorescein staining. In healthy eyes, tear film stability is typically maintained for more than 10 s after eye opening. In dry eye disease (DED), however, TFB is generally observed within 5 s and often appears as dark spots. The primary factors contributing to these abnormalities include mucin layer disruption, inflammation, and decreased tear secretion. Accordingly, several pharmacological agents targeting inflammation and tear film stability, such as rebamipide, diquafosol, cyclosporine, and lifitegrast, have been introduced in clinical practice, in addition to ocular lubricants [3].

Drug repositioning offers several advantages, including a lower risk of failure owing to the established safety profiles of repurposed drugs in both preclinical models and humans, as well as a significant reduction in development time, as most preclinical and safety evaluations have already been completed [4]. One of the most representative successful examples in the ophthalmic field is the repositioning of rebamipide, which was originally developed as a gastroprotective agent, for the treatment of DED. This repositioning is attributed to its ability to promote mucin production and exert protective effects on epithelial tissues. Troxipide (TRO; 3,4,5-trimethoxy-N-(3-piperidyl)benzamide), a gastroprotective agent widely used for the treatment of gastritis and gastric ulcers, has properties similar to those of rebamipide [5]. In addition to its cytoprotective effects, TRO exhibits anti-inflammatory and antioxidant activities. It suppresses neutrophil-mediated inflammation and oxidative stress, increases gastric mucus secretion, and elevates mucopolysaccharide and prostaglandin E2 levels [6,7,8]. Therefore, similarly to rebamipide, TRO is expected to have therapeutic efficacy in the treatment of DED.

Topical eye drop administration is commonly used to treat anterior ocular diseases, including DED. However, a major limitation of the ocular drug delivery system (DDS) is its extremely low bioavailability, often less than 5%, owing to rapid tear turnover, blinking, reflex tearing, nasolacrimal drainage, and the barrier function of the tear film [9,10,11]. To overcome these limitations, eyelid-applied formulations have recently attracted attention. These formulations enable drug transfer into the tear film via application to the eyelids. However, their practical application requires the development of an effective DDS that enhances drug penetration into the eyelids without causing toxicity. Nanoparticles are considered ideal candidates for targeted DDSs because of their small size, high surface area-to-volume ratio, and tunable surface properties [12,13,14]. In ophthalmology, nanosuspensions—defined as colloidal dispersions with particle sizes below 1 μm—have been demonstrated to prolong ocular residence time through interactions between bioadhesive polymers and the ocular mucus layer, while facilitating particle uptake into the mucus layer [15]. For instance, cyclosporine A nanosuspensions have been reported to achieve significantly higher corneal drug concentrations than Restasis^®^ following a single administration [16], along with reduced ocular irritation [17]. Thus, nanomedicine-based ophthalmic DDSs are expected to improve tolerability and patient compliance compared to conventional eye drops, making them a promising strategy for the treatment of anterior ocular diseases, including DED [18,19,20].

The selection of an appropriate animal model is critical for DED research. N-acetylcysteine (NAC) is a mucolytic and reducing agent that cleaves disulfide bonds in mucoproteins, thereby inducing a shift from high- to low-molecular-weight mucin molecules [21]. Previous studies have reported that instillation of 20% NAC into rabbit eyes three times at 5 min intervals results in a histological reduction in the mucin layer covering the cornea and conjunctiva, loss of microvilli, and desquamation of corneal and conjunctival epithelial cells [22,23,24,25,26]. Topical administration of NAC induces mucin degradation on the ocular surface, decreases mucin production, and impairs the mucin retention capacity. Therefore, this mucin-deficient ocular surface model has been widely used to evaluate the mucin-enhancing effects of therapeutic agents on the cornea and conjunctiva.

In this study, we prepared a TRO eyelid gel based on nanoparticle technology and evaluated its therapeutic efficacy against DED using an NAC-induced model.

## 2. Materials and Methods

### 2.1. Animals

Forty-one male Japanese White rabbits (body weight: 2.81 ± 0.50 kg) were obtained from Shimizu Laboratory Supplies Co., Ltd. (Kyoto, Japan). The animals were maintained under controlled environmental conditions, including a 12 h light/12 h dark cycle (lights on from 07:00 to 19:00) and a constant temperature of 25 °C. Standard chow (CR-3; CLEA Japan, Inc., Tokyo, Japan) and water were provided ad libitum. DED was induced by the topical administration of 10% NAC. Subsequently, 1.5% TRO eyelid gels (0.3 g) were applied to the shaved eyelid skin, either as a single dose or repeatedly. In the repeated-treatment protocol, the formulation was administered once daily at 14:00 for six consecutive days. Tear volume, mucin content, TFB time, and ocular surface conditions were evaluated at 18:00. All animal experiments were performed in compliance with internationally recognized guidelines for animal care and use, including the ARRIVE guidelines, the ARVO Statement for the Use of Animals in Ophthalmic and Vision Research, and applicable institutional regulations. Experimental procedures were reviewed and approved by the Institutional Animal Care and Use Committee of Kindai University (approval number: KAPS-2024-009).

### 2.2. Chemicals

TRO, methyl p-hydroxybenzoate, and NAC were procured from FUJIFILM Wako Pure Chemical Corporation (Osaka, Japan). Methylcellulose (MC; SM-4 grade) and carboxypolymethylene (Carbopol; Carbopol^®^ 934) were provided by Shin-Etsu Chemical Co., Ltd. (Tokyo, Japan) and Serva (Heidelberg, Germany), respectively. Schirmer tear test strips were purchased from Showa Yakuhin Kako Co., Ltd. (Tokyo, Japan), and a tear mucin assay kit was obtained from Cosmo Bio Co., Ltd. (Tokyo, Japan). All other reagents and chemicals used in this study were of analytical grade and utilized without further purification unless otherwise specified.

### 2.3. Preparation of Eyelid Gels Containing TRO Nanoparticles

Ophthalmic dispersions containing TRO nanoparticles were prepared as described previously [27,28,29]. Briefly, TRO was mixed with MC in distilled water, and the dispersions were milled at 1500 rpm for 3 h using 0.1 mm zirconia beads and a ShakeMaster^®^ NEO (Biomedical Science, Tokyo, Japan). The milled mixtures were gelled with Carbopol and used as eyelid gels containing TRO nanoparticles (TRO-NP@EG). TRO solid microparticle-based eyelid gels were prepared according to the same protocol without bead mill treatment (TRO-MP@EG). The compositions of TRO-MP@EG and TRO-NP@EG were 1.5% TRO, 0.5% MC, and 3% Carbopol in distilled water (pH 6.8). In this study, whether TRO was successfully nanonized by wet bead milling was evaluated based on changes in particle size distribution and dispersion stability. Dispersion stability was assessed using the following method: aliquots of each sample (2 mL) were placed in 3 mL test tubes and stored at 20 °C under light-protected conditions for 28 days [30,31]. The images of the samples were captured at the end of the storage period (1 month after preparation). In addition, samples were collected from a position 5 mm below the liquid surface, and the dispersibility was evaluated by determining the TRO concentration using high-performance liquid chromatography (HPLC), as outlined below.

### 2.4. HPLC

TRO was measured by HPLC [27,28]. Specifically, TRO was quantified by diluting the prepared samples with methanol. An aliquot of the diluted sample (30 µL) was transferred to a disposable vial, followed by the addition of 100 µL of a methanol-based internal standard solution containing methyl p-hydroxybenzoate. Chromatographic separation was performed using a Shimadzu LabSolutions system (Shimadzu Corp., Kyoto, Japan) with a mobile phase consisting of phosphate buffer and acetonitrile (90:10, *v*/*v*). The column temperature was maintained at 35 °C using a CTO-20AC column oven, and separation was achieved using an Inertsil ODS-3 analytical column (GL Science Co., Inc., Tokyo, Japan). The mobile phase was delivered at a flow rate of 0.25 mL/min, and analytes were detected at a wavelength of 254 nm. The total run time for each analysis was 18 min. A sample volume of 10 µL was injected using a SIL-20AC autosampler. Under these chromatographic conditions, TRO was eluted for approximately 5–6 min, whereas the internal standard was eluted for approximately 14–15 min. The lower detection limit was 25 ng/mL.

### 2.5. Characterization of Eyelid Gels Containing TRO Nanoparticles

Eyelid gels were characterized as described previously [27,28,30,31]. A SALD-7100 laser diffraction nanoparticle-size analyzer (Shimadzu Corp., Kyoto, Japan), with the refractive index set to 1.60-0.10i, was used to measure the size distribution of TRO particles in formulations. The size distribution and number of nanoparticles in the formulation were analyzed using a dynamic light scattering technique (NanoSight LM10; QuantumDesign Japan, Tokyo, Japan). The measurement time was 60 s, and the wavelength and viscosity of the suspension were set to 405 nm (blue) and 1.27 mPa⋅s, respectively. Rheological behavior was evaluated at 20 °C using a tuning-fork oscillation viscometer (A&D Co., Ltd., Tokyo, Japan). The pH of each formulation was determined by the direct application of the samples onto colorimetric pH indicator strips (Merck KGaA; Darmstadt, Germany). The eyelid gel (0.3 g each) was divided into 10 parts, and the TRO content in each part was measured to investigate the dispersity. The solubility of TRO in the formulation was assessed by isolating the dissolved fraction via ultracentrifugation. The dispersions were centrifuged at 100,000× *g* using an Optima™ MAX-XP ultracentrifuge (Beckman Coulter, Osaka, Japan) to separate undissolved solids from the supernatant. The collected supernatant, which corresponded to the equilibrium dissolved TRO concentration, was subsequently quantified using HPLC under the analytical conditions described above. For the physicochemical stability study, TRO@EG was stored for 1 month at 22 °C in a light-protected, tightly sealed container before evaluation.

### 2.6. Drug Release Behavior of TRO@EG

The release behavior of TRO from the prepared TRO@EG was examined using a Franz-type diffusion apparatus equipped with a membrane barrier [27,32]. The receptor compartment was charged with 12.2 mL of a 10 mM phosphate buffer. A membrane filter (MF™-Membrane Filter; Merck Millipore, Tokyo, Japan) with a pore size of 450 nm was mounted between the donor and receptor chambers. Subsequently, 0.3 g of the TEO@EG was carefully placed on the membrane surface. Samples were collected over a 24 h period, and the release profile of TRO was quantified. The particle size distribution of the released nanoparticles and the drug concentration in the receptor phase were analyzed using a NanoSight LM10 and by HPLC, respectively, as described above. This in vitro drug release study was designed to evaluate the release of both dissolved TRO and TRO nanocrystals from the formulation.

### 2.7. TRO Concentration in Rabbit Tear Fluid

TRO@EG (0.3 g) was applied once to each eye. Tear fluid samples were collected at predetermined time points using Schirmer tear test strips. Each strip was immersed in 200 µL of methanol, and TRO was extracted by centrifugation (20,400× *g*, 10 min, 4 °C; TOMY SEIKO Co., Ltd., Tokyo, Japan). The obtained supernatant was used as an analytical sample. The TRO concentrations were quantified using the HPLC method described above. The area under the concentration–time curve from 0 to 180 min (*AUC*_0–180min_) was calculated using the trapezoidal rule. To determine the TRO concentrations in meibum-containing and meibum-free tear fluid, an eyelid speculum was used to prevent contamination of the tear fluid with meibum. Meibum and tear fluid samples were collected 30 and 60 min after the application of TRO@EG (0.3 g) using Schirmer tear test strips [33,34]. Concentrations were measured using the HPLC method described above.

### 2.8. NAC-Induced DED Model

To establish an ocular surface mucin-deficient model, adult male Japanese White rabbits were subjected to repeated topical administration of 10% NAC solution prepared in physiological saline. A fixed volume of 30 µL was instilled into the eye per dose, six times daily at 2 h intervals between 9:00 and 19:00, for two consecutive days. Animals were subjected to subsequent experiments 2 days after the final administration, and this time point was designated as day 0 in the DED model [33,34].

### 2.9. The Evaluation of TRO@EG Efficacy in DED

Topical administration of TRO formulations was initiated after the establishment of the NAC-induced DED model, and this starting point was designated as day 0. The TRO@EG formulation and the commercially available rebamipide ophthalmic suspension, a well-established mucin secretagogue used for the treatment of DED, was administered once daily at 10:00 for five consecutive days. Tear samples were collected 4 h (14:00) after the final administration using Schirmer tear test strips to determine the tear volume and mucin content. Ocular surface imaging was performed 5 days after the initiation of TRO@EG treatment using a DED observation system DR-1 (Kowa Co., Ltd., Aichi, Japan), and tear film stability was evaluated. TFB was assessed as described in [33,34]. TFB area was evaluated at 4 h (14:00) after TRO@EG administration, and the breakup area was quantified 2 s after the final blink using ImageJ software (version 1.54p; National Institutes of Health, Bethesda, MD, USA). Tear volume was determined using Schirmer tear test strips following the administration of TRO formulations. Tear mucin content was quantified using a commercially available enzyme-linked immunosorbent assay (ELISA) kit (Cosmo Bio Co., Ltd., Tokyo, Japan), according to the manufacturer’s protocol. Briefly, tear fluid collected on Schirmer tear test strips was subjected to mucin extraction by immersion in an elution buffer. The extracted samples were analyzed using an ELISA kit and a fluorescence microplate reader (excitation/emission: 336/383 nm) [33,34]. Total mucin content was expressed as µg per eye, and mucin concentration (mg/mL) was calculated by normalizing the total mucin content to the corresponding tear volume.

### 2.10. Statistical Methods

All quantitative data are presented as means ± standard error (S.E.). Statistical analyses were performed using JMP software (version 5.1; SAS Institute Inc., Cary, NC, USA). Comparisons between two groups were conducted using Student’s *t*-test. For experiments involving three or more groups, data were initially analyzed using one-way analysis of variance (ANOVA), followed by the Tukey–Kramer post hoc test for multiple comparisons. A value of *p* < 0.05 was considered to indicate statistical significance.

## 3. Results

### 3.1. Bead-Milling Preparation of Eyelid Gels Containing TRO Nanoparticles

**Figure 1:**
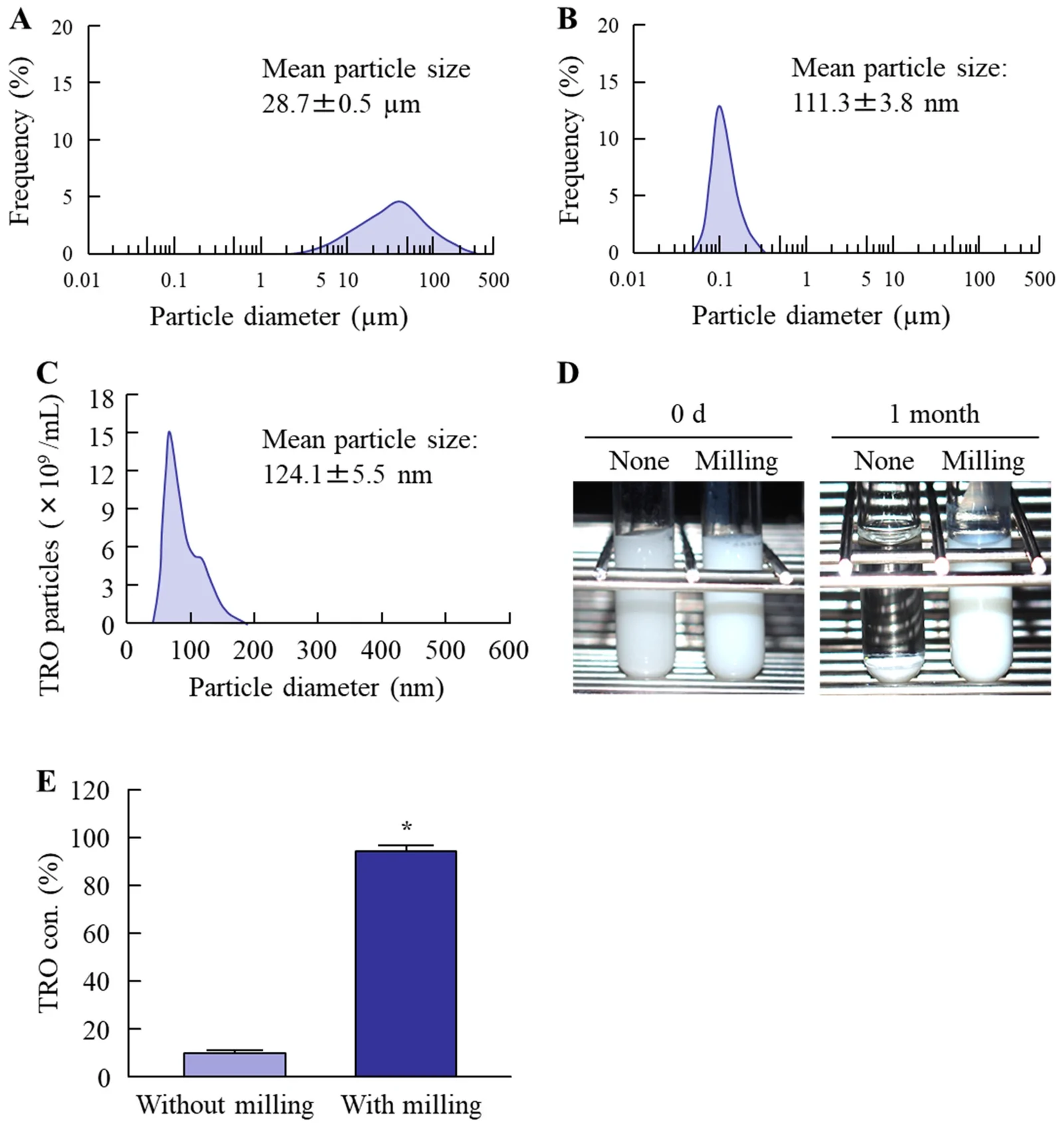
Preparation of TRO nanoparticles by bead-milling treatment. (**A**–**C**) Particle size distribution of TRO dispersions without (**A**) or with bead-milling treatment (**B**,**C**). (**A**,**B**) were measured using a SALD-7100, and (**C**) was measured using a NanoSight LM10. (**D**,**E**) show digital images (**D**) and the dispersion stability (**E**) of TRO particles in dispersions 1 month after bead-milling treatment. n = 6. * *p* < 0.05 vs. TRO-MP@EG for each category. The bead-milling treatment decreased the TRO particle size from 28.9 µm to 111 nm. Moreover, the dispersion stability was enhanced by the bead-milling treatment.

**Figure 2:**
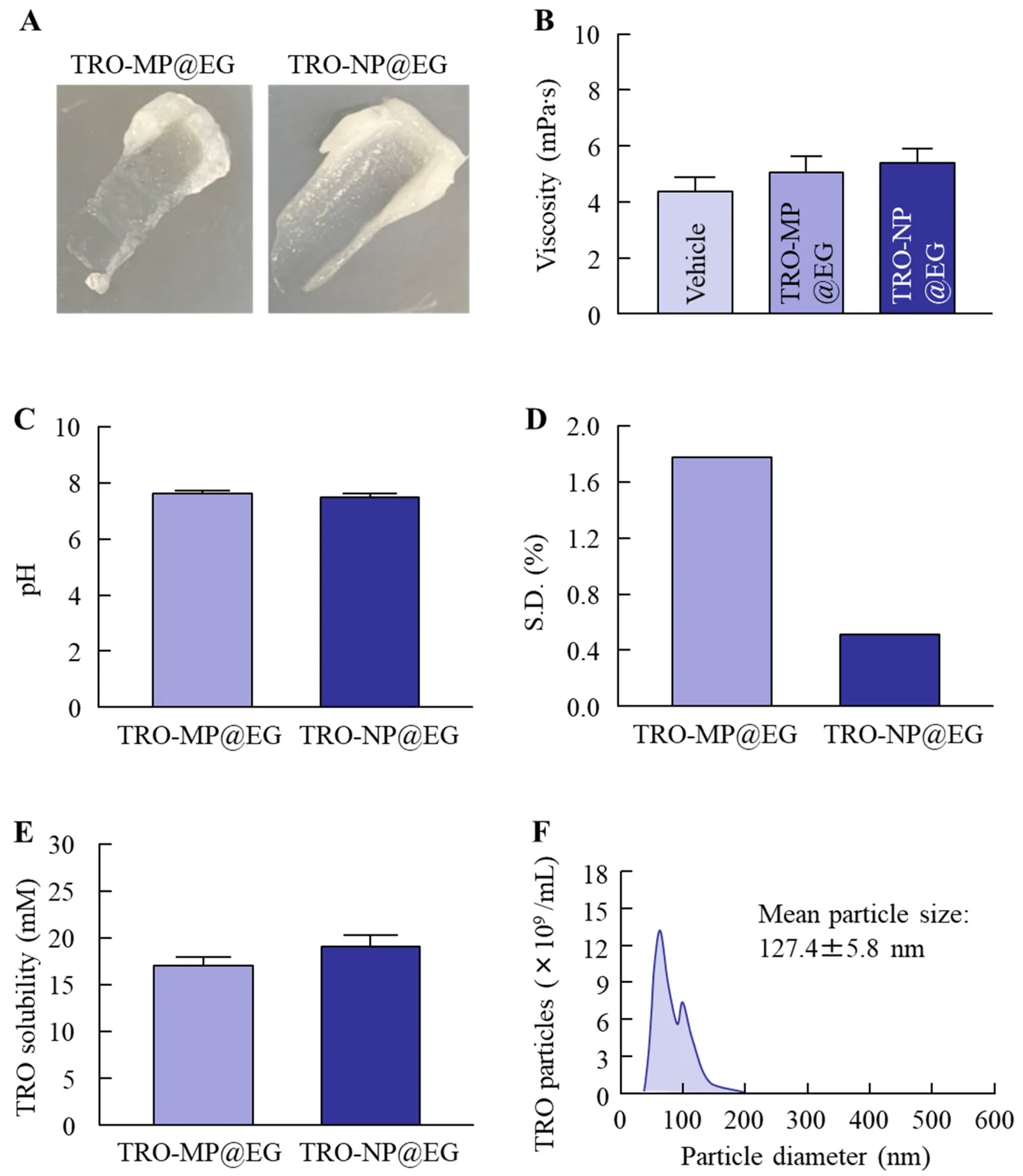
Representative image (**A**), viscosity (**B**), pH (**C**), content uniformity (**D**), and solubility (**E**) of TRO@EG, and particle size distribution of TRO-NP@EG (**F**). n = 6. *p* < 0.05 vs. TRO-MP@EG for each category. The viscosity, pH, and solubility of TRO-MP@EG and TRO-NP@EG did not significantly differ, although the content uniformity of TRO-NP@EG was higher than that of TRO-MP@EG. The drug particles in TRO-NP@EG were in the nanometer size range.

The bead-milling method is a well-known breakdown technique for reducing drug particles to the nanoscale. We first prepared a TRO nanosuspension using bead milling, and subsequently attempted to formulate an eyelid gel based on these nanosuspensions. Figure 1 shows the particle size and changes in the physical state of TRO nanosuspensions during bead milling, and Figure 2 presents the physicochemical properties of the resulting eyelid gel. Following wet bead milling, the particle size of TRO was reduced to approximately 118 nm. Observation of the dispersion revealed improved stability associated with particle size reduction, and high stability was maintained even one month after preparation. Upon gelation, particle aggregates were visible in the TRO-MP@EG, but not in the TRO-NP@EG, which exhibited a smooth, cream-like appearance (Figure 2A). Furthermore, viscosity and pH measurements indicated comparable values between the two formulations, with no significant differences in solubility (Figure 2B,C,E). In contrast, consistent with the visual observations, TRO-NP@EG exhibited less variability within the formulation and demonstrated higher uniformity (Figure 2D). In addition, the mean particle sizes of TRO-MP@EG and TRO-NP@EG were 29.3 ± 1.7 µm and 127.4 ± 5.8 nm, respectively (Figure 2F). In this study, the physicochemical stability of TRO-NP@EG one month after preparation was also evaluated. The mean particle size, drug content, viscosity, and pH were 129 ± 8.1 nm, 1.49 ± 0.1%, 5.5 ± 0.8 mPa·s, and 6.8 ± 0.1, respectively (n = 5), showing no substantial changes compared with those measured immediately after preparation.

### 3.2. Drug Release and Ocular Penetration Behavior Following Eyelid Gel Application

**Figure 3:**
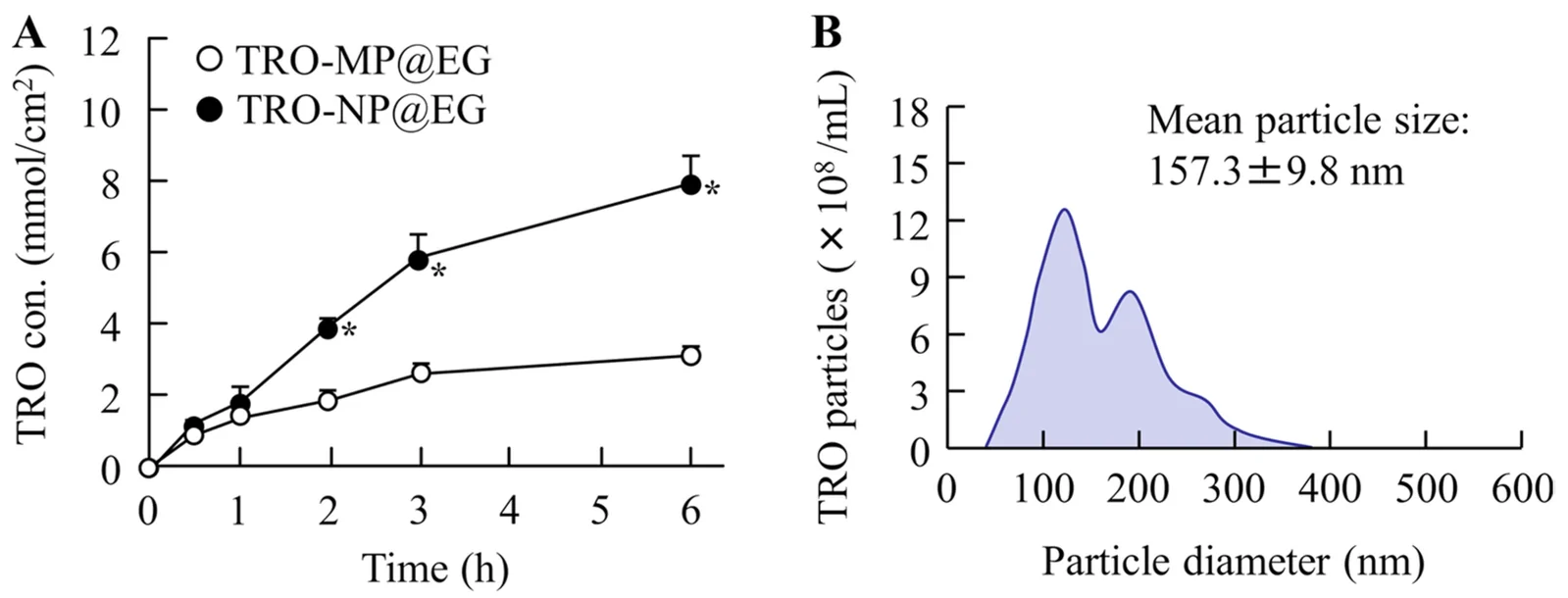
Drug release profile of the eyelid gels (**A**) and size distribution of the released TRO particles (**B**). n = 6. * *p* < 0.05 vs. TRO-MP@EG for each category. The 157 nm TRO particles (mean particle size) were released from TRO-NP@EG, and their TRO release was significantly higher than that of TRO-MP@EG.

**Figure 4:**
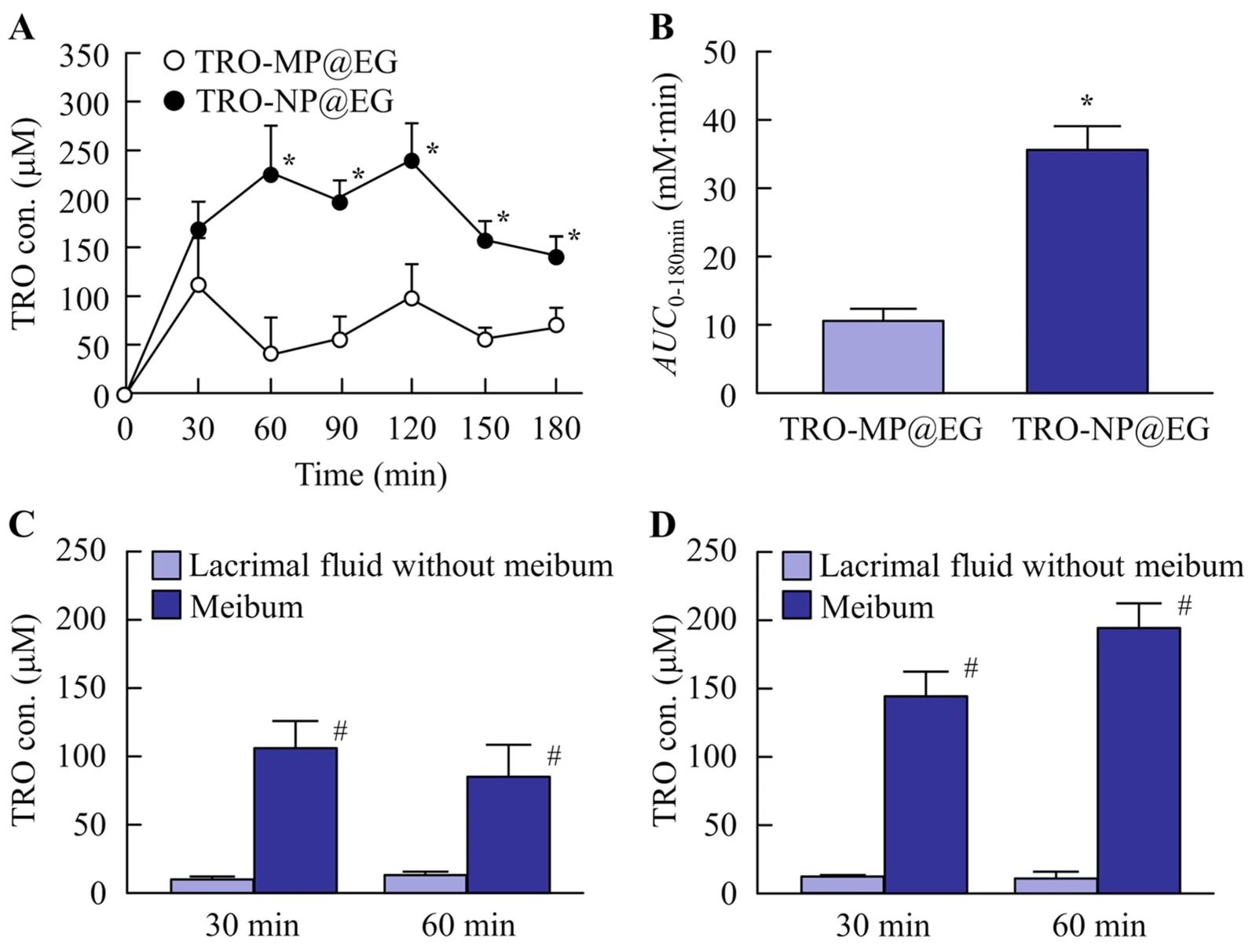
TRO behavior in the ocular surface after the application of eyelid gels. (**A**,**B**) Drug profiles in tear fluid (**A**) and the corresponding AUC 0–180 min levels (**B**). (**C**,**D**) Changes in TRO concentrations in lacrimal fluid without meibum, and meibum at 30 min and 60 min, after the application of TRO-MP@EG (**C**) and TRO-NP@EG (**D**). n = 6. * *p* < 0.05 vs. TRO-MP@EG for each category. ^#^ *p* < 0.05 vs. lacrimal fluid without meibum for each category. TRO levels in the lacrimal fluid in rabbits treated with TRO-NP@EG were significantly higher than those in rabbits treated with TRO-MP@EG, and the TRO was delivered into the tear fluid through a meibum-mediated pathway.

Figure 3 shows the drug release profiles of TRO-MP@EG and TRO-NP@EG using a membrane filter with a pore size of 450 nm. The amount of drug released from TRO-NP@EG at 24 h after the start of the experiment was 2.5-fold higher than that released from TRO-MP@EG. In addition, a portion of TRO released from the gel maintained its solid nanoparticle form, with a mean particle size of 157.6 nm. Next, the amount of TRO transferred into the tear fluid was measured following the application of TRO-MP@EG and TRO-NP@EG to rabbit eyelids (Figure 4A,B). TRO was detected in the tear fluid after application of both eyelid gels. However, the amount of TRO in the tear fluid was significantly higher in the TRO-NP@EG-treated group than in the TRO-MP@EG-treated group. Furthermore, the involvement of the meibum in the transfer of TRO into the tear fluid after the application of TRO-MP@EG and TRO-NP@EG was investigated (Figure 4C,D). High levels of TRO were detected in the meibum of both eyelid gels, whereas transdermal penetration not mediated by meibum was minimal. Moreover, the transfer of TRO into the meibum in the TRO-NP@EG-treated group was significantly higher than in the TRO-MP@EG-treated group. Furthermore, we investigated the physical state of TRO in the meibum secreted from the meibomian glands in this study. The results revealed that no solid (crystalline) TRO was detected, indicating that TRO was present in its dissolved form.

### 3.3. Therapeutic Effects of Eyelid Gel Application in a DED Rabbit Model

**Figure 5:**
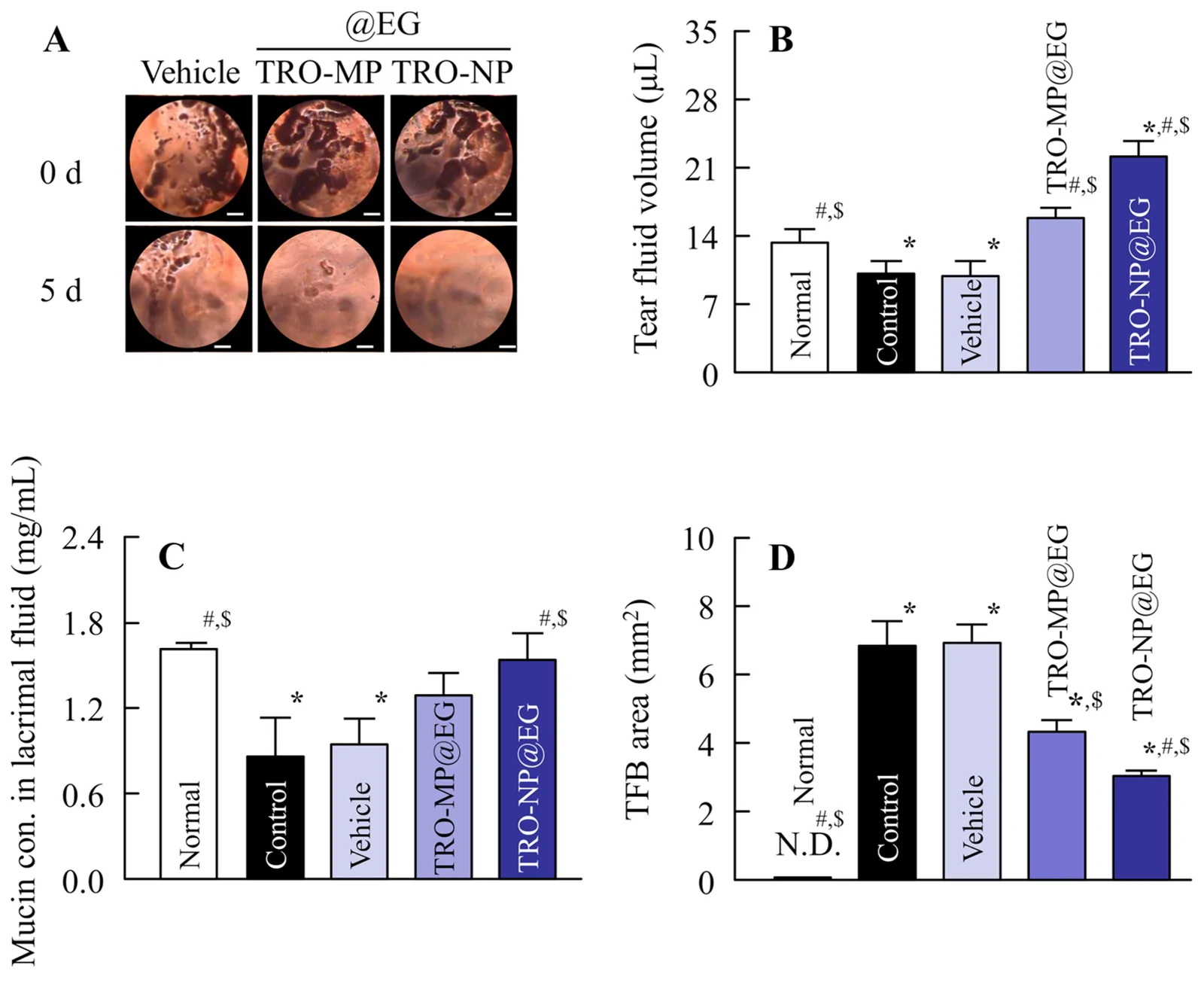
Changes in digital images (**A**), levels of tear fluid volume (**B**), mucin in lacrimal fluid (**C**), and TFB area (**D**) in the DED rabbit model treated with eyelid gels. n = 6. * *p* < 0.05 vs. normal rabbit (Normal) for each category. ^#^ *p* < 0.05 vs. non-treated DED rabbit model (Control) for each category. ^$^ *p* < 0.05 vs. vehicle-treated DED rabbit model (Vehicle) for each category. The treatment of TRO-NP@EG improved the degraded tear fluid volume, mucin content, and TFB levels.

Figure 5 shows the therapeutic effects of TRO-MP@EG and TRO-NP@EG on DED. Compared to normal conditions, a decrease in mucin levels in the tear fluid was observed in the NAC-administered DED model. In addition, the tear volume was reduced, and the TFB area increased. In contrast, treatment with TRO@EG improved the reduction in mucin levels and increased the tear volume. Furthermore, the TFB area significantly decreased compared to that in the control and vehicle groups. Moreover, a comparative evaluation of the therapeutic effects of TRO-MP@EG and TRO-NP@EG demonstrated that the TRO-NP@EG-treated group exhibited superior efficacy to the TRO-MP@EG-treated group in terms of mucin levels, tear volume, and TFB area. Furthermore, the TFB area in rabbit instilled with the commercially available rebamipide ophthalmic suspension was 4.1 ± 0.5 mm^2^ (n = 5), indicating that TRO-NP@EG showed a significantly higher therapeutic effect. (Figure 5D). Throughout the treatment period, no visible signs of ocular or periocular toxicity, including eyelid erythema, corneal damage, conjunctival hyperemia, or other observable ocular abnormalities, were detected. This supports preliminary tolerability but does not establish the safety profile of the formulation. Further studies are needed to elucidate the safety profile.

## 4. Discussion

**Figure 6:**
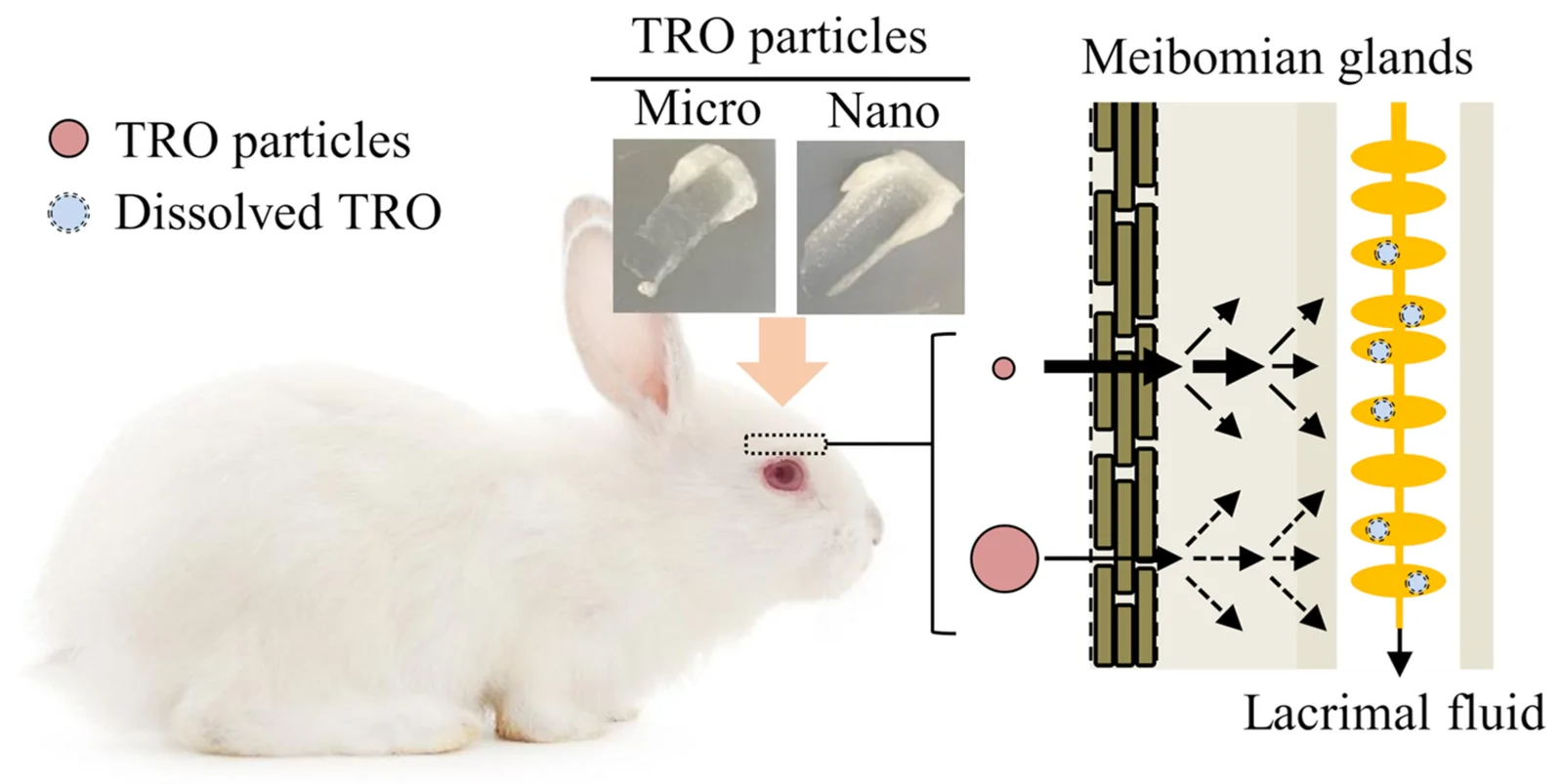
Proposed schematic illustration of drug transport from the eyelid-applied gel containing micro- and nanoparticles to the tear fluid.

Topical drug application to the eyelids has attracted increasing attention as a novel route for ocular therapy, as it is expected to provide an improved duration of pharmacological effects compared with conventional eye drop administration. However, it is essential to incorporate technologies that enhance transdermal absorption to achieve sufficient drug permeation into the eyelids and its subsequent delivery to the tear fluid. In recent years, nanotechnology has garnered considerable interest as a strategy to improve the pharmacological efficacy of ophthalmic formulations. We have previously reported that reducing drug particles to the nanoscale enhances transdermal permeability [28,32,34]. In the present study, we focused on TRO, a gastroprotective agent, and attempted to prepare a TRO eyelid gel containing nanoparticles (TRO-NP@EG) with an average size of approximately 100 nm. In addition, we explored its potential for the treatment of DED, as well as its applicability as an eyelid-applied formulation. As a result, topical application of TRO@EG to the eyelid enabled the DDS to enter the tear fluid via the meibum. Furthermore, the DDS exhibited high retention on the ocular surface and demonstrated superior therapeutic efficacy in an NAC-induced DED model (Figure 6).

Several approaches have been reported for the preparation of solid drug nanoparticles based on “breakdown” and “build-up” processes, and we have previously described a bead-milling method using additives [27,28,29,35,36]. Among these, wet bead milling in the presence of additives, such as MC, is particularly effective for producing high-quality nanosuspensions. Furthermore, we have reported that TRO can be successfully reduced to the nanoscale using this method [27,28,29]. In the present study, we first prepared TRO nanosuspensions based on our established method and subsequently gelled the formulation to allow its application as an eyelid preparation. The physicochemical characterization confirmed the successful preparation of TRO nanocrystals by wet bead milling (Figure 1). Next, to develop an eyelid gel, these nanoparticles were incorporated into the gel. Carbopol, a type of hydrogel, offers advantages such as high safety and ease of removal [37,38,39]. In addition, owing to its water solubility, it can retain poorly soluble drug nanoparticles within the base without solubilization and is suitable for releasing intact nanoparticles from the formulation [28,32,34]. Based on these considerations, Carbopol was selected as the base for the eyelid gel. The prepared TRO-NP@EG exhibited higher dispersion stability in suspension than TRO-MP@EG, and a uniform distribution of TRO within the gel was confirmed. Furthermore, based on solubility, approximately two-thirds of TRO in the eyelid gel was present as solid nanoparticles within the gel. Other physicochemical properties, such as viscosity and pH, were comparable between the formulations, indicating that TRO-MP@EG could serve as an appropriate control for TRO-NP@EG. In this study, the release of TRO particles from TRO@EG was evaluated using a membrane filter and a Franz diffusion cell (Figure 3). The results indicated that TRO-NP@EG is stable and exhibits high drug release, and that TRO is released from the gel base as solid nanoparticles.

Having successfully developed an eyelid gel capable of releasing TRO as solid nanoparticles, we next investigated the drug delivery pathway following the application of TRO-NP@EG to the eyelids of rabbits (Figure 4). As a result, TRO-NP@EG enhanced trans-eyelid delivery of TRO and prolonged its retention in the tear fluid compared with TRO-MP@EG (Figure 4A,B). In addition, TRO was not detected in the tear fluid at 1 h after the instillation of either TRO micro- or nano-suspensions; however, when TRO-NP@EG was applied to the eyelids, TRO remained detectable even at 3 h post-application (Figure 4A,B). The eyelid differs structurally from other skin tissues, as its inner side uniquely contains meibomian glands. These glands contain lipid components known as meibum, which are secreted into the tear fluid through the glandular orifices to prevent tear evaporation [40]. Based on this anatomical feature, the involvement of the meibum in the transfer of TRO from TRO@EG into the tear fluid was investigated (Figure 4C,D). As shown in Figure 2E, the solubility of TRO in the eyelid gel did not differ significantly between TRO-MP@EG and TRO-NP@EG. Consistent with this finding, no significant difference in the amount of drug released from the gels was observed at 1 h (Figure 3A). In contrast, marked differences in the amounts of TRO detected in the meibum and tissues were observed within 1 h after administration (Figure 4). These findings suggest that the enhanced delivery is unlikely to be explained solely by the permeation of dissolved TRO into the eyelid and subsequent distribution into the meibum. Rather, we speculate that the nanoparticles released from the gel play a major role in enhancing drug delivery through the meibomian gland pathway. Thus, high concentrations of TRO were detected in the meibum (lipid fraction): this suggests that, although a portion of the penetrated TRO passed directly through the eyelid into the tear fluid, the majority was first incorporated into the meibum and subsequently transferred to the tear fluid. Moreover, no solid TRO was detected, indicating that TRO was present in its dissolved form. Because meibum is lipid-rich, it exhibits a high affinity for hydrophobic drugs such as TRO. Therefore, the solubility and strong affinity of the poorly soluble TRO for meibum likely contributed to these findings. Rabbits possess numerous hair follicles that provide openings to the skin, which may facilitate nanoparticle penetration. Therefore, nanoparticle permeation in rabbits may be higher than that in humans. Further studies, such as fluorescence imaging and histological analyses, are required to elucidate the delivery mechanism of TRO-NP@EG in the human eyelid tissue.

Demonstrating the therapeutic potential of TRO-NP@EG requires validation of its efficacy using an appropriate DED model. Therefore, the selection of a suitable animal model is critical. NAC is widely known to cleave disulfide bonds in mucoproteins, leading to a shift toward lower-molecular-weight mucin species. Topical administration of NAC induces histological alterations in the mucin layer covering the corneal and conjunctival surfaces, including depletion of mucin components, loss of microvilli, desquamation of epithelial cells, and reduction in tear film thickness. In contrast, rebamipide treatment increases the conjunctival goblet cell density in normal rabbits and enhances mucin-like substance levels in NAC-treated models. Collectively, these findings support the suitability of the NAC-induced rabbit model for evaluating mucin-related therapeutic interventions. Based on this rationale, the therapeutic efficacy of TRO-NP@EG was investigated in this model. DED is characterized by a reduction in tear volume and mucin secretion on the ocular surface. Mucin plays an essential role in maintaining tear film stability, facilitating smooth blinking through lubrication, preserving optical surface integrity, providing a barrier against external insults, and contributing to the clearance of pathogens and debris. DED is a multifactorial disorder involving tear film and ocular surface abnormalities. In the present study, tear volume and mucin content were assessed following the application of TRO-NP@EG. Treatment with TRO-NP@EG restored the mucin levels that were reduced by NAC exposure (Figure 5C). This result supports the previous finding that TRO increases mucus secretion and elevates mucopolysaccharide levels, thereby reinforcing the mucosal barrier function [5,6,7,8]. In addition, an increase in tear secretion and a decrease in tear film instability were observed (Figure 5C,D), accompanied by normalization of ocular surface conditions (Figure 5A). Interestingly, tear production exceeded the baseline levels observed under normal conditions. This phenomenon may reflect a compensatory increase in tear secretion in response to ocular surface impairment induced by NAC, which persisted transiently even after the restoration of the mucin layer following TRO-NP@EG treatment. Based on these observations, we propose a mechanistic hypothesis in which TRO-NP@EG is applied to the eyelids, penetrates the skin, and is subsequently taken up by the meibomian glands. The drug is then secreted into the tear film along with the meibum, where it exerts pharmacological effects on the ocular surface mucosa, ultimately improving ocular surface abnormalities. It has been reported that TRO suppresses inflammatory and oxidant activities as well as neutrophil-mediated damage, while increasing gastric mucus secretion and elevating mucopolysaccharide and prostaglandin E2 levels [6,7,8]. Although these mechanisms were not directly investigated in the present study, they may collectively explain the enhanced therapeutic efficacy observed following eyelid administration of TRO-NP@EG. Further mechanistic studies evaluating goblet cell density, mucin expression (e.g., MUC5AC and membrane-associated mucins), inflammatory cytokines, oxidative stress markers, and epithelial barrier function will be necessary to confirm these hypotheses. In addition, we compared the therapeutic efficacy of our formulation with the commercially available rebamipide ophthalmic suspension, a well-established mucin secretagogue used for the treatment of DED, as a positive control. When the commercially available rebamipide suspension was administered once daily for five consecutive days under the same experimental conditions used for the eyelid formulation, the TFB area was 4.1 ± 0.5 mm^2^ (n = 5), indicating that TRO-NP@EG exhibited greater therapeutic efficacy (Figure 5D). These findings indicate that the use of TRO-NP@EG is a promising therapeutic strategy for DED.

## 5. Conclusions

We designed a solid nanoparticle-based eyelid gel containing TRO (TRO-NP@EG) and demonstrated its ability to sustainably deliver troxipide to the ocular surface, potentially through the meibomian glands. In addition, local administration of TRO@EG improved the therapeutic outcomes in an NAC-induced DED model, and TRO-NP@EG markedly enhanced these effects. These findings provide important insights into a novel delivery pathway via the meibomian glands, and demonstrate its superior therapeutic efficacy against DED, which may be useful for designing future studies aimed at treating DED. On the other hand, although the rabbit is a well-established animal model for ophthalmic research, several anatomical and physiological differences from humans should be considered when interpreting the present findings. Rabbits have a relatively higher hair follicle density, which may influence follicular drug penetration following eyelid application. In addition, species differences in eyelid skin structure and barrier function may affect transdermal drug permeability. Furthermore, the morphology, distribution, and function of the meibomian glands differ between rabbits and humans, which could alter drug delivery to the target tissue and influence therapeutic efficacy. Therefore, caution is warranted when extrapolating the present results directly to human patients. Further investigations using human eyelid tissues for local tolerability, penetration, and therapeutic efficacy, and ultimately clinical studies, will be required to validate the translational potential of this eyelid drug delivery strategy. Moreover, further investigation is warranted to elucidate the trans-eyelid delivery mechanism for TRO nanoparticles. Given previous reports suggesting the involvement of endocytic pathways in nanoparticle transport [41,42,43], ongoing studies are examining the effects of endocytosis inhibitors to elucidate their roles in eyelid penetration and the subsequent migration of TRO nanoparticles into the meibomian glands.

## Data Availability

The original contributions of this study are included in the article material. Further inquiries can be directed to the corresponding author.

## Abbreviations

BAC	Benzalkonium chloride
DED	Dry eye disease
EG	Eyelid gel
MC	Methylcellulose
NAC	N-acetylcysteine
ODDS	Ocular drug-delivery system
TRO	Troxipide
TRO-MP	TRO microsuspension
TRO-NP	TRO nanosuspension
S.E.	Standard error
SEM	Scanning electron microscopy
SPM	Scanning probe microscopy
TFB	Tear film breakup
TG-DTA	Thermogravimetry and differential thermal analysis
XRD	X-ray diffractometry
